# Draft genome of the brown-rot fungus *Fomitopsis pinicola* GR9-4

**DOI:** 10.1016/j.dib.2017.09.043

**Published:** 2017-09-25

**Authors:** Reddy Prakash Kancherla, Mikael Brandström Durling, Jan Stenlid, Nils Högberg

**Affiliations:** Department of Forest Mycology and Plant Pathology, Swedish University of Agricultural Sciences, Uppsala, Sweden

**Keywords:** Draft genome, Brown-rot, Fomitopsis

## Abstract

Basidiomycete brown-rot fungi have a huge importance for wood decomposition and thus the global carbon cycle. Here, we present the genome sequence of *Fomitopsis pinicola* GR9-4 which represent different *F. pinicola* clade than the previously sequenced North American isolate FP-58527 SS1. The genome was sequenced by using a paired-end sequence library of Illumina and a 2.5k and 5k mate-pair library (ABI SOLiD). The final assembly adds up to a size of 45 Mb (including gaps between contigs), with a GC-content of 56%. The gene prediction resulted in 13,888 gene models. The genome sequence will be used as a basis for understanding population genomics, genome-wide association studies and wood decay mechanisms of this brown-rot fungus.

**Specifications table**

**Table:** 

Subject area	Biology
More specific subject area	Mycology, Genomics
Type of data	Genomic sequence, gene annotation and phylogenetic position of *F. pinicola* GR9-4
How data was acquired	Whole-genome sequencing using the Illumina TruSeq PE and ABI SOLiD platform at SNP&SEQ Technology Platform of Uppsala University Hospital.
Data format	Raw sequencing reads, draft genome assembly and gene prediction.
Experimental factors	Spores were collected from fruiting bodies of *F. pinicola* on spruce logs and DNA was extracted from single monokaryon isolate.
Experimental features	Sequencing reads were assembled using ABySS/1.3.6 and BWA-mem v0.7.4
Data source location	Samples were collected in 2012 from spruce logs, Granåsan, Sweden.
Data accessibility	NCBI (BioProject: PRJNA354689), GenBank assembly accession: GCA_001931775.1, Contigs: MPVS01000001–MPVS01002178. (https://www.ncbi.nlm.nih.gov/Traces/wgs/?val=MPVS01#contigs)

**Value of the data**

• *F. pinicola* plays an important role in the carbon cycle of conifer forests as the species is one of the most prominent wood decayers in forest ecosystems, but the biology of this fungus is poorly studied at the population level.
• Draft genome assembly of *F. pinicola* will increase the knowledge of the biochemical process of wood degradation and create an opportunity for comparative studies with other brown-rot fungi.

## 1 Data

**Fig. 1:**
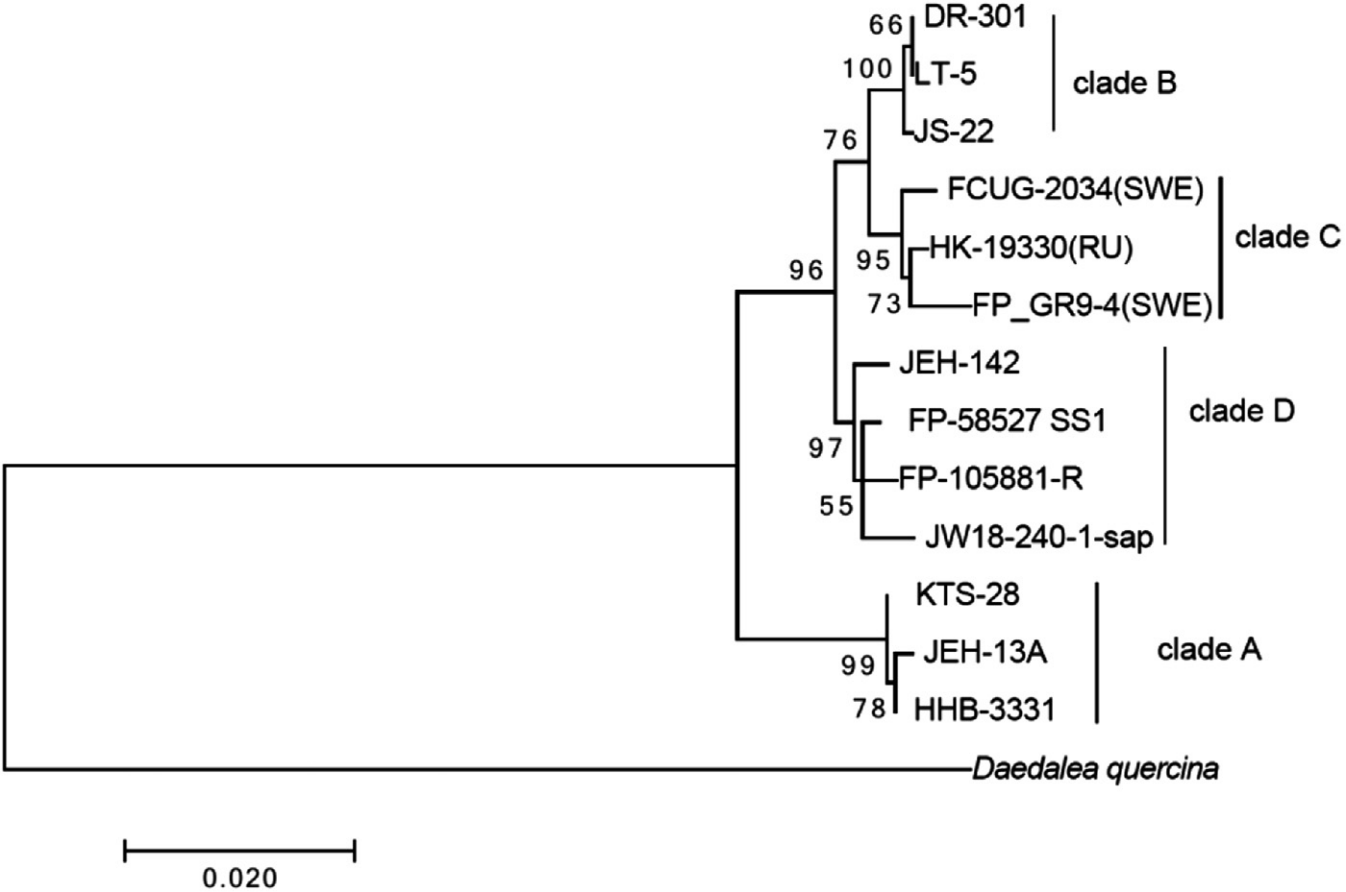
Maximum likelihood tree of 1357 base pair, partitioned sequence of ITS, EF1A and RPB2 from a representative sample of 13 samples of *F. pinicola*. Numbers below internodes denote bootstrap support based on 1000 replicates. Clade A, D, and B refer to North America and clade C refer to Europe. The tree is rooted to *Daedalea quercina* as outgroup.

We present the draft genome assembly and gene prediction of the fungus *Fomitopsis pinicola,* an important and ubiquitous brown-rot fungus of boreal forests causing a cubical brown-rot in both softwood and hardwood [1]. The broad host range and phenotypic differences with respect to color and form, already observed by Fries [2], led to speculation that cryptic species might be present. Early findings based on crossings of single spore isolates of *F. pinicola* confirmed intersterility among lineages within North America and between North America and Europe [3]. Recently a multi-locus phylogenetic study confirmed that *F. pinicola* is a species complex encompassed of four well-supported phylogenetic species, three in North America and one in Eurasia [4]. The genome of *F. pinicola* GR9-4 was sequenced in order to provide a basis for population genomics, transcriptomics and genome-wide association studies. The final assembly contained 1920 contigs larger than 1000 bp (an N_50_ contig length of 69,460 bp a N_80_ contig length of 18,102 bp), with a largest length of 788,230 bp, which were assembled into 1613 scaffolds larger than 1000 bp in size and the maximum length being of 1,100,126 bp. In total, the genome sequence adds up to a size of 45 Mb (including gaps between contigs), with a GC-content of 56%. The sequencing read coverage depth of the total assembly was 127-fold. The gene prediction resulted in 13,888 gene models (Table 2). The draft genome assembly information of *F. pinicola* compares well to other sequenced genomes within the Agarimycotina [5]. Genes with predicted functions for plant polysaccharide (cellulose, hemicellulose and pectin) degradation involved in the breakdown or modification of glycoconjugates were identified in the genome. We found 403 carbohydrate-active enzymes (CAZymes) using the pipeline dbCAN [13] with E-values below 1e-4. These were divided into 209 Glycoside Hydrolases (GHs), 81 Glycosyl Transferases (GTs), 5 Polysaccharide Lyases (PLs) and 108 Carbohydrate Esterases (CEs). In addition to the CAZymes, 51 redox enzymes that act in conjunction with CAZymes (AAs) and 48 Carbohydrate-Binding Modules (CBMs) were found. The CAZymes-coding genes profiles for *F. pinicola* GR9-4 was similar to the profile of the North American isolate *F. pinicola* FP-58527 SS1, but their secondary metabolite profiles differed substantially as analyzed by the antiSMASH 3.0 genome mining of biosynthetic gene clusters [14]. The next generation sequencing data is available from the NCBI under GenBank assembly accession GCA_001931775.1. We also applied a maximum likelihood analysis (Fig. 1) to infer the phylogeny of the three nuclear genes ITS, EF1A and RPB2 respectively from a representative collection of isolates within the species complex, using *Daedalea quercina* as an outgroup [3], [5], [6], analyzed in MEGA 7 [7]. This analysis was performed in order to reveal the phylogenetic position of the two sequenced *F. pinicola* genomes within the four well supported clades; European clade-C which is distinctly separated from North American clades-A, B & D. Our sequenced isolate, GR9-4, belongs to *F. pinicola* clade C while the previously sequenced North American isolate, *F. pinicola* FP-58527 SS1 belongs to the distinctly different *F. pinicola* clade D. This difference will permit future estimations of positive selection to the divergence between species. Furthermore, information about the draft genome sequence of *F. pinicola* GR9-4 will be helpful for future studies in population genomics and genome-wide association in order to reveal the wood decay mechanisms of this brown-rot fungus.

**Table 2:** Genome features of *F. pinicola* GR9-4.

Features	*F. pinicola* (GR9-4)
Assembled length	45,195,986
Contig length	44,555,307 (98.5%)
Sequencing read coverage depth (fold)	127.7
Gap length	640,679 (1.4%)
Number of scaffolds (>1 kb)	1613 (100%)
Number of contigs (>1 kb)	1920 (99.5%)
GC-Content	55.5%
No. of predicted genes	13,888
No. of evidence-supported genes	13,016
Average length of transcripts	1912.14
Average coding length	1385.96
Total exons	82,309
Average exons per transcript	5.93

**Table 1:** Data used in assembly and scaffolding. PE, paired-end; ME, mate-pair.

Library type	No. reads (pairs)	Mean Insert size
Illumina (PE)	30,690,310	329
SOLiD (ME) 2.5K	323,417,186	1873
SOLiD (ME) 5K	315,004,134	4369

## 2 Experimental design, materials and methods

### 2.1 Library

• Strategy: Whole-genome DNA sequencing.
• Taxon: *Fomitopsis pinicola.*
• Sample details: spores were restrained on a glass side placed below living sporocarps for overnight and single germinated spores were collected under dissecting microscope to produce single monokaryon isolates.
• Location: Granåsen*,* Sweden.
• Sample handling: The sample was collected in 2012 by Reddy Prakash Kancherla and Nils Högberg and stored at 4 °C at the Department of Forest Mycology and Plant Pathology, Swedish University of Agricultural Sciences, Sweden.
• Library: Paired-end 100 bp reads and Mate-pair 2.5 kb and 5 kb reads.

### 2.2 Library construction protocol

Basidiospores were restrained on a glass slide placed below living sporocarps for overnight. Serially diluted spores were spread on Hagem-agar petri dishes - sterilized medium containing of 0.5 g each of NH_4_NO_3,_ KH_2_PO_4_ and MgSO_4_·7H_2_O, 5 g of glucose, 5 g of malt and 10 g of agar in 1 L of deionized water. Three to five days after inoculation, between 1 and 20 single germinated spores were collected under dissecting microscope and cultured on fresh media to produce single monokaryon isolates. Monokaryon isolates were cultured on liquid Hagem medium for 3 weeks. DNA was extracted by standard CTAB protocol. 3 μg DNA was used for sequencing on the Illumina platform and 5 μg was used for mate-pair library from ABI SOLiD (SNP&SEQ Technology Platform, Uppsala University). The raw sequencing data produced is shown in Table 1.

### 2.3 Data processing

The obtained reads were assembled, scaffolded, gaps were filled and validated, by using ABySS/1.3.6 [8], SSPACE-LongRead [9], GapCloser-bin-v1.12-r6 [10] and BWA-mem v0.7.4 [11]. The draft genome sequence was annotated using the MAKER pipeline [12] using transcripts and gene catalogues from other basidiomycete fungi as evidence.
